# Rumor Has It…: Relay Communication of Stress Cues in Plants

**DOI:** 10.1371/journal.pone.0023625

**Published:** 2011-11-02

**Authors:** Omer Falik, Yonat Mordoch, Lydia Quansah, Aaron Fait, Ariel Novoplansky

**Affiliations:** 1 Mitrani Department of Desert Ecology, Blaustein Institutes for Desert Research, Ben-Gurion University of the Negev, Midreshet Ben-Gurion, Israel; 2 The French Associates Institute for Agriculture and Biotechnology of Dryland, Blaustein Institutes for Desert Research, Ben-Gurion University of the Negev, Midreshet Ben-Gurion, Israel; French National Centre for Scientific Research, Université Paris-Sud, France

## Abstract

Recent evidence demonstrates that plants are able not only to perceive and adaptively respond to external information but also to anticipate forthcoming hazards and stresses. Here, we tested the hypothesis that unstressed plants are able to respond to stress cues emitted from their abiotically-stressed neighbors and in turn induce stress responses in additional unstressed plants located further away from the stressed plants. *Pisum sativum* plants were subjected to drought while neighboring rows of five unstressed plants on both sides, with which they could exchange different cue combinations. On one side, the stressed plant and its unstressed neighbors did not share their rooting volumes (UNSHARED) and thus were limited to shoot communication. On its other side, the stressed plant shared one of its rooting volumes with its nearest unstressed neighbor and all plants shared their rooting volumes with their immediate neighbors (SHARED), allowing both root and shoot communication. Fifteen minutes following drought induction, significant stomatal closure was observed in both the stressed plants and their nearest unstressed SHARED neighbors, and within one hour, all SHARED neighbors closed their stomata. Stomatal closure was not observed in the UNSHARED neighbors. The results demonstrate that unstressed plants are able to perceive and respond to stress cues emitted by the roots of their drought-stressed neighbors and, via ‘relay cuing’, elicit stress responses in further unstressed plants. Further work is underway to study the underlying mechanisms of this new mode of plant communication and its possible adaptive implications for the anticipation of forthcoming abiotic stresses by plants.

## Introduction

Signal perception, learning and decision-making abilities are usually thought to rely on sophisticated central nervous systems (CNS); however, information acquisition and communication are ubiquitous even among the oldest and most rudimentary life forms [1]–[4]. Plants are able to perceive and adaptively respond to their environment based on subtle biotic and abiotic signals and cues [4]–[8]. Recent evidence demonstrates that plants are also able to communicate with both allies and foes [6], [7], [9]–[12]. For example, following local stress or damage, plants not only increase local resistance and defense, but also induce defensive responses in remote organs of the same plant [13]–[15]. In response to herbivory, some plants release volatile organic compounds (VOC) that attract natural enemies of their herbivores [reviewed in 10], induce chemical defenses in their undamaged neighbours [e.g. 16], and prime them to respond more readily and intensely to subsequent herbivore attacks [12], [17], [18]. Belowground signaling has been demonstrated to both affect plant interactions with diverse soil micro- and macro-organisms [19] and to intricately mediate competitive interactions between plants [11], [20], [21].

Here, we studied the possibility that long-range communication of stress cues is mediated by the perception and emission of stress cues by unstressed plants. Specifically, we tested whether unstressed plants are able to perceive and respond to stress cues emitted by their drought-stressed neighbours, and whether induced unstressed plants also emitted stress cues, which in turn further elicit stress responses in additional unstressed plants. Additionally, we studied whether the drought stress cues are communicated above- and/or below-ground.

## Materials and Methods

### Experimental design

**Figure 1:**
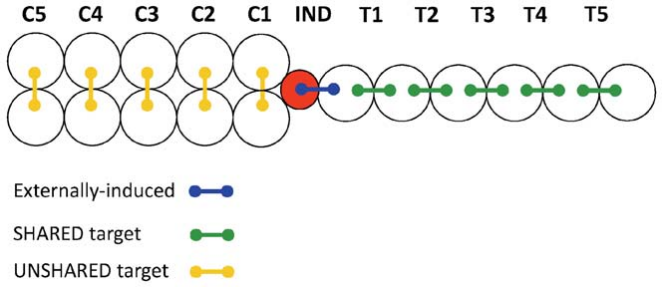
Testing for stress cuing - the experimental setup. Circles represent rooting receptacles and connector lines represent split-root plants. Plants neighbouring the externally-induced plant (IND) either shared (SHARED; T1–T5) or did not share (UNSHARED; C1–C5) their rooting volumes with their immediate neighbours. External induction was carried out by injecting either water (control) or mannitol (osmotic stress) to the red rooting receptacle. Stomatal width was destructively measured in different experimental sets immediately before (0 min), and 15 and 60 minutes after the red receptacle was injected with either water or mannitol.

Split-root *Pisum sativum* var. Dunn plants were subjected to osmotic stress while neighbouring rows of five unstressed on both sides (IND; Fig. 1). Each plant had two similarly-sized roots (split-root plants), which were grown in either exclusive (UNSHARED) receptacles, or while sharing (SHARED) their rooting receptacles with their neighbours. On one of its sides, the stressed IND plant shared one of its rooting receptacles with its nearest neighbour (T1), which was the first in a row of five plants, which shared their rooting receptacles with their immediate neighbours (SHARED; T1–T5; Fig. 1). This configuration allowed the SHARED plants to both perceive stress cues from the IND plant and exchange amongst themselves both root exudates and volatile cues. On the other side of the stressed IND plant, a row of neighbouring plants did not share their rooting receptacles with their neighbours (UNSHARED; Fig. 1), which limited their potential communication to volatile cuing.

Osmotic stress was inflicted upon one of the roots of the IND plant using mannitol - a natural sugar-alcohol osmoticum whose addition to the rooting medium is commonly used to elicit controlled drought in higher plants [e.g. 22]. Responses to osmotic stress and stress cues were tested by recording stomatal aperture of the IND and its neighbours, following the addition of mannitol to the rooting receptacle of the root of the IND plant which was not shared by its neighbours (red receptacle; Fig. 1). Comparing between the responses of the SHARED and UNSHARED target neighbours provided indications as to the involvement of shoot and root cuing in the communication of stress cues.

To account for possible confounding effects of plant handling on stomatal aperture, control treatments were added where the IND plants were injected with water (Fig. 1).

### Experimental setup

The plants were grown so that they developed two equal roots following removal of the tip of the seminal root (‘split-root plants’), [23]. Three days from germination, the seminal root was severed two mm below the hypocotyl and the plants were replanted in damp vermiculite. Seven days from germination, the stump of the seminal root typically regenerated three lateral roots that were thinned down to two roots. Plants with two symmetric 25–30 mm long roots were planted so each of their roots was grown in a separate 50 mL, 30 mm diameter plastic receptacle (Greiner, Frickenhausen, Germany), filled with distilled water. To ensure identical distances between adjacent plants, despite the different positional arrangements of the rooting receptacles of the SHARED and UNSHARED plants, a 15 mm diameter (15 ml) receptacle was used in the position of injection (red receptacle in Fig. 1). This measure ensured that potential volatile cues traveled the same distance from the IND plant towards both its SHARED and UNSHARED neighbours. The rooting receptacles were secured to each other using plastic soldering. The top of each receptacle was tightly covered by paraffin film (Parafilm, Chicago, IL, USA). The openings through which the roots of the IND and T1 plants penetrated the injected receptacle (red receptacle in Fig. 1) were minimal in size and sealed by petroleum jelly to prevent the possibility of capillary migration of mannitol from the injected to the T1 receptacle. The same procedure was used in the water treatments, to control for possible confounding effects of the petroleum jelly on the experimental plants.

### Chemical analysis and bioassay

In order to rule out the possibility that mannitol was transferred from the injected receptacle to the roots of the neighbouring target plants, either via seeping through the petroleum jelly barrier or via root uptake and exudation, the rooting media of the target receptacles nearest to the IND receptacle (shared by the roots of the IND and T1 plants) and the rooting receptacle shared by plants T4 and the T5 were analyzed for the presence of mannitol using gas chromatography-mass spectrometry (GC-MS) immediately upon the termination of the experiment. The rooting media were cryo-lyophilized in a Freezmobile II concentrator (Virtis Co., Inc., Gardiner, NY, USA). Each lyophilized sample was dissolved in 500 µL MeOH and the internal ribitol quantification standard was added. The samples were then dried in vacuum overnight. Following drying, residues were re-dissolved in a mixture of 40 µL of 20 mg mL^−1^ methoxyamine hydrochloride and pyridine, and were derivatized for 2 h at 37°C, followed by 30 min in a retention-time standard mixture of 70 µL MSTFA (N-methyl-N-[trimethylsilyl]trifluoroacetamide) and 8 µL of 0.02 v/v alkanes dissolved in pyridine. All samples were analyzed for the presence of mannitol following Lisec *et al.* (2006) [24], using GC-MS (Thermo Scientific, Waltham, MA, USA, and the extraction and analysis protocols routinely used for the analyses of polar compounds [25]. Absolute mannitol concentrations were determined by comparison with calibration standard curve response ratios of various concentrations of standard substance solutions, including the internal standard ribitol [26]. Standard mannitol was ran in a dilution series, ranging from 1.25 ng to 100 ng of injected substance (1 µL injection volume) and identified using Xcalibur software (Finnigan, Thermo Scientific, Waltham, MA, USA). Response ratios relative to the internal standard ribitol were calculated, and linear correlations between the response ratio and the amount of the substance were determined. The GC-MS clearly detected mannitol concentrations as low as 3.02×10^−9^ M.

### Growth conditions and induction protocol

The plants were grown in a growth chamber, at 25°C, under continuous 130 µE m^−2^ sec^−1^ of cool-white fluorescent light, for seven days, before they were treated with either mannitol or water. Throughout this period, distilled water was supplemented (injected through the paraffin film) as needed to ensure that the roots were immersed in water.

External induction was carried out by pumping 7.5 mL of water from the induction receptacle (red receptacle, Fig. 1) and injecting 7.5 mL of either distilled water (water controls) or 0.8 M mannitol (final concentration in root medium of 0.4 M; Sigma, St. Louis, MO, USA).

In order to test whether the minimal concentration of mannitol traceable by the GC-MS analyses could induce stress responses in plants, a bioassay was conducted in which *Pisum* plants were subjected to 0 (water controls) or 3.02×10^−7^ M mannitol (i.e. 100-fold the minimal mannitol concentration clearly detectable by the GC-MS; 10 replications per treatment) and their average stomatal apertures were estimated 60 min after induction. Plant sizes, growth conditions, induction procedure and estimation of stomatal aperture were conducted using the same protocols as described for the main experiment.

### Stomata measurements and plant performance

Stomatal aperture was used as a highly sensitive phenotypic expression of plant response to osmotic stress [27]. Stomatal aperture was estimated by measuring the average width of stomata immediately before the external induction (0 min; water treatment only), and 15 and 60 minutes following the external induction (in both water and mannitol treatments).

Stomatal aperture was estimated from epidermal impressions following Sachs et al. 1993 [28]: the lower surfaces of 1–2 fully-unfurled 20–30 mm^2^ leaflets of each sampled plant were copied using a fresh mixture of Vinyl Polysiloxane dental impression material (Elite HD+, Badia Polesine, Rovigo, Italy). Following hardening, the resulted imprints were further copied with clear nail polish, which resulted in transparent preparations suitable for microscopic examination. Because the preparation of the imprints was disruptive, each plant set (depicted in Fig. 1) was only measured once, i.e. separate replication sets were sampled at different times and water and mannitol treatments.

**Figure 2:**
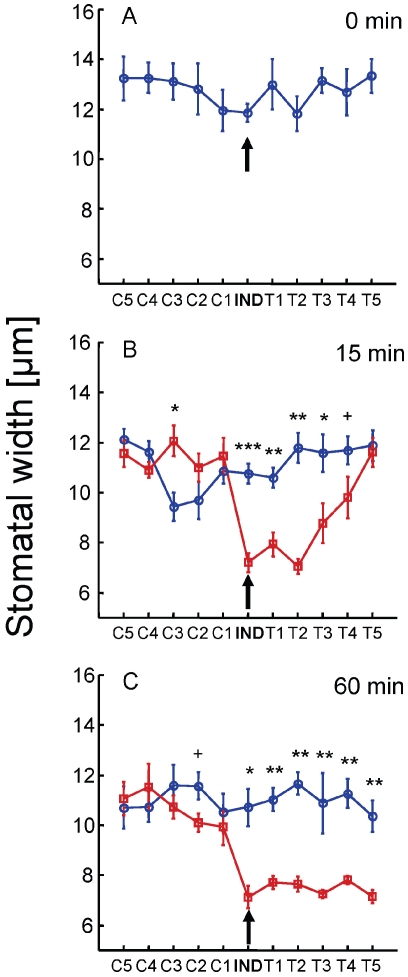
Stomatal responses to stress and communicated stress cues. Stomatal width of induced plants (pointed at by black arrows) and their SHARED (T1–T5) and UNSHARED (C1–C5) neighbours immediately before (0 min; A), 15 (B) and 60 (C) minutes after one of the roots of the IND plant, was injected with either water (blue) or mannitol (red). Data represent means ±1 s.e.m.; N = 6. ***: p<0.001; **: p<0.01; *: p<0.05; +: 0.05<p<0.1.

Stomata measurements were carried out using AxioVision software (Carl Zeiss MicroImaging, Thornwood, NY, USA) on digital images of the nail-polish preparations. Average stomatal width was calculated from the data of at least 10 stomata per plant, selected haphazardly from 2–5 0.02 mm^2^ areas in the centre of each microscopic preparation. Accordingly, each data point (Fig. 2) represents the average width of at least 60 stomata nested within six replication sets (N = 6) per treatment per time interval.

The effects of the experimental stress induction on plant performance were tested on additional experimental sets, which were harvested 14 d after mannitol and water induction. Upon harvest, plants were separated to root and shoot parts, and their dry biomasses were estimated after drying in a ventilated oven at 60°C for at least 72 hours.

In order to avoid observer bias, all samples were handled and analyzed using a single-blind protocol, whereby the observer could not know the identity of the samples.

The significance of treatment main effects (water versus mannitol) was analyzed using one-way ANOVAs and within-treatment kinetics was analyzed using repeated-measures ANOVAs (SYSTAT 10; [29]). Mann-Whitney U tests were used to evaluate the differences between the effects of water and Mannitol on individual IND, T(1–5) and C(1–5) plants.

Similar results were obtained from four independent repeats of the experiment and only the results from the last run are presented here.

### Results and Discussion

Fifteen minutes following water injection to the unshared rooting volume of the IND plant (red receptacle; Fig. 1), stomatal width of all plants decreased by an average of 13% (F = 22.4; *p*<0.001), compared to their state before the injection, and no significant changes were recorded over the subsequent 45 minutes (F = 0.1; *p* = 0.74, Fig. 2). This slight, though consistent, stomatal closure reflected a response to the physical handing of the plants, and thus served as a baseline for the comparison of plant responses to the stress treatment. Fifteen minutes after mannitol injection, the IND plant and its two nearest SHARED neighbours (T1, T2) closed their stomata by 39% compared to their water controls (F = 116.8, *p*<0.001), while SHARED target neighbours positioned further away from the IND plant (T3–T5) maintained increasingly opened stomata (Fig. 2B). Sixty minutes after the mannitol injection, the width of the stomata of all the SHARED plants was drastically reduced to a similar extent (Fig. 2C). In contrast, 15 minutes after the mannitol injection, the stomata of the UNSHARED (C1–C5) plants remained opened to a similar extent as their water controls (Fig. 2B; F = 1.1, *p* = 0.293). Sixty minutes after the mannitol injection, the stomatal apertures of the UNSHARED plants nearest the IND plant (C1–C2) were non-significantly different from their aperture 15 min after the injection; however stomatal aperture of the C2 plants were 9.6% smaller than their water controls (Fig. 2C).

**Figure 3:**
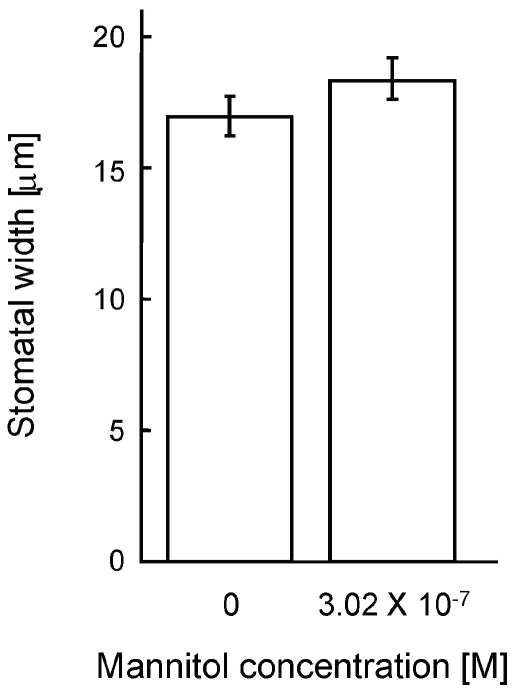
Testing the validity of GC-MS quantification of mannitol. Stomatal width of plants, the roots of which have been subjected to 0 (water controls) or 3.02×10^−7^ M mannitol, showing that even 100-fold the minimal mannitol concentration detectable by GC-MS analyses did not evoke stomatal closure in the experimental plants.

In order to rule out the possibility that mannitol was transferred from the injected receptacle to the roots of the neighbouring target plants, either via seeping through the petroleum jelly barrier or via root uptake and exudation, the rooting media of the target receptacles nearest to the IND receptacle (shared by the roots of the IND and T1 plants) and the rooting receptacle shared by plants T4 and the T5 were analyzed for the presence of mannitol immediately upon the termination of the experiment. GC-MS analysis demonstrated no traces of mannitol down to a concentration of 3.02×10^−9^ M in any of the tested samples. Although such low mannitol concentrations are not known to elicit stomatal closure, further testing was conducted to ascertain that such mannitol concentrations could not induce stomatal closure in the studied plants. Exposing the roots of *Pisum* plants to either distilled water (controls) or to a 3.02×10^−7^ M mannitol solution (100-fold greater than the lowest mannitol concentration decisively traceable by the GC-MS analyses), demonstrated no significant differences in stomatal aperture (Fig. 3). The GC-MS analyses and the results from the bioassay demonstrated that the stomatal closure observed in the T1–T5 SHARED target neighbours (Fig. 2B) reflected true communication amongst the IND and the SHARED neighbours and could not have resulted by either artifact sipping or active transfer of mannitol from the injected (red receptacle; Fig. 1) to the rooting receptacles of the target plants.

The fact that the IND plants rapidly closed their stomata following a direct exposure to an osmotic stress is neither novel nor surprising [e.g. 30]. However, the results also demonstrated that unstressed plants were able to perceive and respond to stress cues emitted by the roots of their stressed neighbours. The gradual, yet rapid, response of increasingly distant SHARED, but not of UNSHARED target plants, demonstrated that the observed communication was chiefly, if not solely, conducted between neighbouring roots rather than amongst shoots. Furthermore, the stomatal closure in the remote unstressed target neighbours (T2–T5) show that unstressed plants are not only able to perceive and respond to stress cues emitted by their stressed neighbours, but that they also release stress cues which can be perceived by additional unstressed plants, creating *a cascading chain of stress responses* in plants that are positioned increasingly further away from the stressed plants.

**Figure 4:**
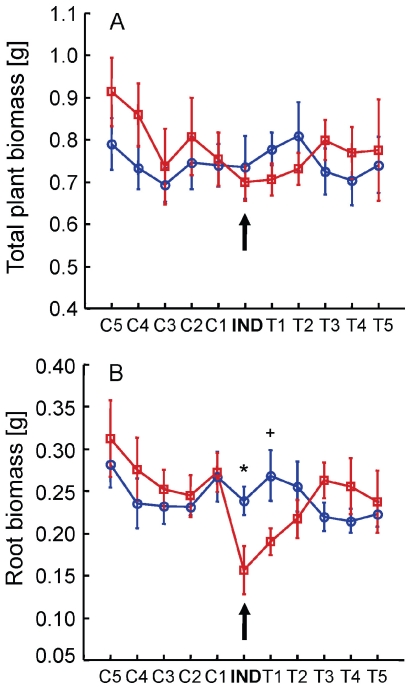
Plant performance following stress induction and the perception of communicated stress cues. Total plant dry biomass (A) and root dry biomass (B) of induced plants (pointed at by black arrows) and their SHARED (T1–T5) and UNSHARED (C1–C5) neighbours, 14 days after one of the roots of the IND plant was injected with either water (blue) or mannitol (red). Data represent means ±1 s.e.m.; N = 5. *: p<0.05; +: 0.05<p<0.1.

The described drought induction resulted in limited long term effects on the growth of the IND and target plants. Fourteen days after mannitol injection, no significant effects were found on the total biomass of the IND, T(1–5) and C (1–5) plants (Fig. 4A). Interestingly, root biomass was 35 and 29% lower in the IND and T1 plants, respectively; compared to their water controls (Fig. 4B), implying decreased root allocation in these plants compared to their water controls.

The results suggest that unstressed plants are able to “eavesdrop” on their osmotically-stressed neighbours and respond in ways that might prepare them for subsequent stress; however, further work is needed to study the longer-term priming effects of such stress cues on the unstressed target plants. This presented communication between the IND and the T1 plants is comparable to the communication between herbivore-damaged plants and their undamaged neighbours [12], [31], [32]. However, the results also demonstrate a novel feature of plant-plant communication: elicited by their stressed neighbours, unstressed plants not only exhibit stress response but also emit stress cues that are perceived by additional unstressed plants. Although not directly demonstrated, recent evidence suggests that the described chain-communication might exist and play an adaptive role in other plant systems. For example, green leaf volatiles (GLV) and jasmonates were shown to be involved in both herbivore repellency [7], [12] and volatile induction of defenses in undamaged neighbouring plants [33]–[35]. Although the precise mechanisms enabling these phenomena are still unclear, some evidence suggests that both jasmonates and GLV might induce each other's or even their own biosynthesis and activity [e.g. 36], [37]. Much like in the case of root stress cuing (Fig. 2), such a system might involve positive-feedbacks, which both enhance defense responses of the affected plants and induce chain-communication, whereby VOC emitted by damaged plants induce an increased production of defensive VOC in their neighbours, which in turn elicit defense responses in additional undamaged plants.

At this early stage, the selective advantage and the ecological implications of the described responses are still uncertain and require further studying; however, the reduced root growth in T1 SAHRED plants might support the hypothesis that responses to communicated osmotic stress cues might include longer term priming, conferring adaptation to subsequent osmotic stresses.

It is expected that cue-emitting plants might bear the costs of the production and emission of costly metabolites, and possibly more importantly, the competitive costs involved in the emission of warning cues that might be utilized by their neighbouring competitors [38]. Accordingly, such “information leakiness” may be understood in terms of the inability of damaged or stressed plants to avoid the emission of compounds that are subsequently perceived by their neighbours. Although this interpretation cannot be dismissed, given that unstressed plants were as affective as their stressed neighbours in inducing stress responses in additional unstressed neighbours (Fig. 2), it is unlikely to fully explain the evolution of the observed stress cuing. An arguably more plausible, although not-mutually exclusive, rationale for the emission of stress cues might be based on the selective advantage conferred by the warning of remote organs on the same plant [39], members of the same-clone [40] and kin [41]. To be evolutionary stable, the advantage of emitting such warning signals must outweigh its accompanying costs, which is less likely to occur in plants whose signals are highly generic and thus perceivable by competitors [12]. Accordingly, external cuing of osmotic stress cues and other ecologically-relevant information is expected to be more prevalent in large plants, where external signaling among organs of the same plant might increase the effectiveness and speed of damage or stress warning [39], in sectorial plants, where the lack of physiological integration limits or totally prevents internal communication [31], and - due to kin-selection [41] - in clonal and other plants whose kin or clone-mates are spatially aggregated [42], [43]. Regardless of the selective advantage rendered to stress-cue emitters, plastic responsiveness of unstressed plants to stress cues is potentially advantageous as it might allow plants anticipate forthcoming stress [4] while avoiding the potentially heavy costs involved in continuous non-plastic stress tolerance [44]. However, the adaptive value of such plastic responses is expected to strongly depend on the reliability of the stress cues and thus to positively correlate with the tightness of the correlation between the presence of anticipatory stress cues and the materialization of subsequent stressful events [11]. Accordingly, it is hypothesized that such anticipatory responses are more common in plants that live where bouts of water shortage are followed by longer or more severe life-threatening droughts, or where plants grow along predictable spatial soil-water gradients created in and around seasonal aquatic habitats [e.g. 45].

Although the underlying mechanisms for the presented results are still unknown, the results do provide a few clues as to the nature of the involved cues. Specifically, the results suggest that the stress signals are- a) produced under osmotic stress, b) readily emitted by the roots of osmotically-stressed plants, c) perceived by plant roots, and d) involved in stress response, including stomatal closure, regardless of the osmotic status of the plant. A plant hormone that satisfies all of these requirements is abscisic acid (ABA; [e.g. 46]–[49], whose involvement in the described phenomena is currently studied.

### Conclusions

The reported results suggest that plants might be able to communicate underground stress cues and respond to various environmental challenges in ways that have been traditionally attributed to higher organisms. However, rather than implying advanced coordinated networking of the types found in social birds and mammals, the results demonstrate the existence of a simpler type of networking, whereby apparent coordination might hinge on information leakiness and neighbour eavesdropping, such as in some cases of cross-taxon alarm cuing and eavesdropping against predators [50]–[52].

Further work in underway, aiming at the mechanisms and adaptive implications of the observed communication of stress cues. Special attention is given to the possibility, which was demonstrated in the case of insect herbivory [12], that the perception of early abiotic stress cues both primes unstressed plants to better tolerate later stress events and renders performance costs in primed plants which are not subjected to subsequent abiotic stress.
